# Kinetic Simulation of Lipid Oxidation and Lycopene Antioxidant Activity in Tomato Pomace Using ANSYS Chemkin

**DOI:** 10.3390/foods15091522

**Published:** 2026-04-28

**Authors:** Lucian Dordai, Adrian Vasile Timar, Lacrimioara Senila, Lucian Cuibus, Anca Becze

**Affiliations:** 1 National Institute for Research and Development of Optoelectronics INOE 2000, Research Institute for Analytical Instrumentation, 67 Donath Str., 400293 Cluj-Napoca, Romania; lucian.dordai@icia.ro (L.D.); lacri.senila@icia.ro (L.S.); 2 Faculty of Environmental Protection, University of Oradea, 1 Universității Str., 410087 Oradea, Romania; atimar@uoradea.ro; 3 Faculty of Food Science and Technology, University of Agricultural Sciences and Veterinary Medicine Cluj-Napoca, 400372 Cluj-Napoca, Romania

**Keywords:** tomato pomace, lipid oxidation kinetics, lycopene stability, radical scavenging, hexanal, ANSYS Chemkin, reaction mechanism, linoleic acid, thermal processing

## Abstract

This study investigates the kinetics of lipid oxidation and the antioxidant activity of lycopene in tomato pomace using a combined computational–experimental approach. A reaction mechanism describing initiation, propagation, hydroperoxide formation, and radical scavenging was implemented in ANSYS Chemkin 2025 R2 and simulated under controlled conditions at 50, 70, and 90 °C for up to 12 h. The model was validated using experimental measurements of linoleic acid, lycopene, and hexanal obtained from thermally treated tomato pomace. The results showed a strong temperature dependence of oxidation processes, with minimal changes at 50 °C and a transition to a propagation-dominated regime at 90 °C. Linoleic acid degradation reached 18.17% after 12 h at 90 °C, accompanied by a significant increase in hexanal formation, while lycopene loss remained below 5%. The model accurately reproduced experimental trends, with high correlation coefficients (R^2^ = 0.9761 for linoleic acid, 0.9899 for lycopene, and 0.9982 for hexanal). Hydroperoxides were identified as key intermediates, accumulating prior to decomposition into volatile products. The results demonstrate that the proposed kinetic model provides a reliable tool for predicting lipid oxidation behavior in tomato by-products and highlights the critical influence of temperature on oxidative stability. Mean percentage errors ranged from 10.03% (hexanal) to 23.52% (linoleic acid), consistent with the complexity of the matrix.

## 1. Introduction

Tomato pomace is an abundant agro-industrial by-product generated during the processing of tomatoes for juice, paste, and canned products [1,2]. Globally, millions of tons of tomato pomace are produced annually, yet a substantial fraction remains underutilized or is disposed of as waste [1,3]. This material is rich in bioactive compounds, particularly lycopene, beta-carotene, tocopherols, and polyunsaturated fatty acids (PUFAs), most notably linoleic acid, which is concentrated in the seed fraction [4,5,6]. The valorization of tomato pomace as a functional food ingredient, nutraceutical source, or encapsulation matrix has attracted considerable research interest in recent years [1,2]. However, the chemical instability of its lipid and carotenoid components under thermal processing conditions represents a major challenge to industrial application and product quality assurance [4].

Lipid oxidation is one of the primary deterioration pathways in PUFA-rich food matrices. The autoxidation mechanism follows a well-established free radical chain process involving initiation, propagation, and termination stages [7]. During initiation, reactive oxygen species abstract hydrogen atoms from bis-allylic positions in polyunsaturated fatty acids such as linoleic acid, generating lipid radicals [8,9]. These radicals rapidly react with molecular oxygen to form peroxyl radicals (ROO•), which then abstract hydrogen from adjacent lipid molecules, propagating the chain reaction and generating hydroperoxides (ROOH) as primary oxidation products [7,8,9,10]. The thermal decomposition of hydroperoxides leads to secondary oxidation products, including volatile aldehydes such as hexanal, which are important markers of oxidative deterioration and contribute to off-flavor development in processed foods [11]. The rate and extent of these reactions are strongly dependent on temperature, oxygen availability, and the presence of antioxidant compounds [7,10]. Lycopene, the predominant carotenoid in tomato pomace, has been extensively studied for its antioxidant properties [12,13]. As a highly conjugated polyene, lycopene is an efficient quencher of singlet oxygen and a scavenger of peroxyl radicals, exerting a protective effect on lipid oxidation [14]. Lycopene is itself susceptible to thermal isomerization and oxidative degradation, particularly at temperatures above 70 °C [15]. The balance between the radical-scavenging role of lycopene and its own thermal stability is therefore a critical determinant of oxidative quality in tomato-derived products subjected to thermal treatments. Despite a substantial body of experimental literature on lycopene stability and lipid oxidation in tomato products, the mechanistic interplay between these two processes at the kinetic level remains incompletely characterized [16,17].

Kinetic modeling of lipid oxidation has emerged as a powerful tool for predicting the shelf life and quality evolution of lipid-containing foods [18]. Approaches based on elementary reaction mechanisms, including Arrhenius-type temperature dependence, have been successfully applied to model autoxidation in bulk oils and emulsified systems [19,20]. Computational fluid dynamics and reaction engineering software such as ANSYS Chemkin, originally developed for combustion chemistry, have more recently been adapted to simulate complex reaction networks in food-relevant systems, owing to their capacity to handle stiff systems of ordinary differential equations and coupled multi-step mechanisms [21,22]. The application of Chemkin-based kinetic simulation to food by-product oxidation, and specifically to the coupled lipid oxidation–antioxidant degradation system in tomato pomace, has not been previously reported. A critical gap in the existing literature concerns the absence of validated predictive kinetic models capable of simultaneously describing linoleic acid degradation, lycopene antioxidant consumption, and hexanal formation in tomato pomace under industrially relevant thermal conditions. Most published studies have addressed these processes in isolation or have relied on empirical degradation kinetics, without incorporating mechanistic reaction steps or intermediate species dynamics [19,20]. Furthermore, the role of hydroperoxides as transient intermediates linking primary and secondary oxidation has rarely been quantified experimentally or modeled explicitly in tomato-based matrices. This mechanistic gap limits the ability to predict and control oxidative quality during thermal drying, pasteurization, or storage of tomato pomace-derived ingredients.

The aim of this study was to develop, parameterize, and rigorously validate a mechanistic, multi-step kinetic model capable of quantitatively describing the coupled processes of lipid autoxidation and carotenoid-mediated radical scavenging in tomato pomace under thermally induced conditions relevant to industrial food processing. Specifically, the study sought to: (i) construct and implement an elementary reaction network in ANSYS Chemkin integrating initiation, propagation, hydroperoxide accumulation–decomposition, and dual antioxidant pathways of lycopene, (ii) resolve the temperature-dependent, non-linear dynamics of radical intermediates, oxygen consumption, and secondary oxidation products across kinetically distinct regimes, (iii) elucidate the mechanistic role of transient species, particularly hydroperoxides, as critical nodes linking primary lipid degradation to volatile formation, and (iv) quantitatively assess the predictive robustness of the model through multi-parameter experimental validation against linoleic acid degradation, lycopene depletion, and hexanal generation. The study aims to bridge the gap between idealized homogeneous kinetic modeling and structurally constrained food matrices by critically evaluating model deviations arising from matrix-induced diffusional limitations and lipid compartmentalization. By integrating computational reaction engineering with targeted experimental validation, this work advances beyond empirical kinetic descriptions toward a predictive, mechanism-driven framework that supports process optimization, oxidative stability control, and rational valorization of tomato pomace within circular food systems.

From a food technology perspective, controlling lipid oxidation during thermal processing is essential for preserving the nutritional and sensory quality of tomato by-products intended for further valorization. The present model is therefore designed not as a purely theoretical framework, but as a predictive tool to support the optimization of processing parameters such as temperature and residence time, with direct implications for the production of stable, bioactive-rich ingredients from tomato pomace.

## 2. Materials and Methods

### 2.1. Kinetic Simulation of Lipid Oxidation and Lycopene Antioxidant Activity

A kinetic modeling approach was developed to investigate the oxidation behavior of lipids and the antioxidant activity of lycopene under conditions relevant to food processing and storage. The simulation framework enables the prediction of radical formation, propagation dynamics, and secondary oxidation products in a controlled reaction environment. The oxidation kinetics of linoleic acid and the radical scavenging activity of lycopene were simulated using ANSYS Chemkin 2025 R2 (ANSYS Inc., Canonsburg, PA, USA). Simulations were performed using the Closed Homogeneous Reactor (batch reactor) model, which assumes a perfectly mixed system with uniform temperature and species distribution. This approach eliminates spatial gradients and is appropriate for evaluating intrinsic chemical kinetics in simplified lipid oxidation systems. Although tomato pomace is a heterogeneous solid matrix, the gas-phase homogeneous reactor approximation has been previously employed in food lipid oxidation modeling to decouple intrinsic reaction kinetics from mass-transfer effects [23].

This approach is considered valid for establishing baseline kinetic parameters, with the understanding that diffusional limitations within the real matrix will cause systematic deviations, particularly at intermediate temperatures, as discussed in Section 4. The reactor was operated under constant temperature and constant volume conditions. The reactor volume was fixed at 100 cm^3^, and the system pressure was maintained at 1 atm. Gas-phase behavior was described using the ideal gas law. Each simulation was conducted over a total reaction time of 12 h to allow sufficient progression of primary and secondary oxidation reactions. To assess the influence of thermal conditions, simulations were performed at 50 °C, 70 °C, and 90 °C, corresponding to typical processing encountered in food systems.

#### 2.1.1. Initial Reactant Composition

The initial composition of the reaction system was defined in terms of mole fractions. Oxygen was included to simulate oxidative conditions, while nitrogen served as an inert carrier gas. Linoleic acid was selected as a representative polyunsaturated fatty acid, and lycopene was incorporated as a model antioxidant compound. All radical intermediates and secondary products were initialized at zero concentration (Table 1).

#### 2.1.2. Reaction Mechanism

A simplified reaction mechanism was constructed to describe the autoxidation of linoleic acid and the antioxidant activity of lycopene. The mechanism includes elementary steps corresponding to initiation, propagation, hydroperoxide formation and decomposition, and radical scavenging reactions. The kinetic parameters (Arrhenius constants) were derived from literature data on lipid autoxidation, hydroperoxide decomposition, and antioxidant radical scavenging reactions, and were further fine-tuned within physically meaningful ranges to reproduce the experimental trends observed in tomato pomace [24,25,26,27,28,29] (Table 2).

#### 2.1.3. Thermodynamic Data

Thermodynamic properties for all species were defined using NASA polynomial coefficients over the temperature range of 300–5000 K [30]. These polynomials provide temperature-dependent values of heat capacity, enthalpy, and entropy required for solving the kinetic model. The thermodynamic database included the following species: LINOLEIC, LINRAD, O_2_, HO_2_, ROO, ROOH, HEXANAL, FRAG12, N_2_, LYCOPENE, and LYCORAD.

#### 2.1.4. Numerical Solution

The temporal evolution of species concentrations was obtained by solving the system of coupled ordinary differential equations (ODEs) derived from the reaction mechanism. The Chemkin solver integrates these equations to generate concentration profiles for reactants, intermediates, and products over the simulation period.

### 2.2. Model Validation Approach

The experimental design was developed to validate the kinetic simulation results obtained using ANSYS Chemkin. The measured concentrations of linoleic acid, lycopene, and hexanal under controlled thermal conditions were compared with the concentration profiles predicted by the kinetic model. This combined experimental–computational approach allowed the evaluation of the accuracy of the proposed reaction mechanism and its ability to reproduce temperature-dependent oxidation processes occurring in tomato pomace.

#### 2.2.1. Sample Treatment

Fresh tomato pomace samples were obtained from a local tomato processing facility immediately after industrial processing. The samples were transported to the laboratory and analyzed without prior storage in order to preserve their original chemical composition. To reproduce the thermal conditions used in the kinetic simulations and to experimentally validate the predictive capability of the model, samples were subjected to controlled heating in a Memmert UFF400 laboratory oven (Memmert GmbH + Co. KG, Schwabach, Germany). The tomato pomace was introduced into the oven only after the set temperature had been reached and stabilized within the chamber, ensuring consistent and reproducible thermal exposure. Thermal treatments were performed at 50 °C, 70 °C, and 90 °C, corresponding to the temperatures used in the Chemkin simulations. Samples were analyzed after 4 h and 12 h of heating, and fresh untreated samples (0 h) were analyzed as controls. The purpose of this experimental design was to compare the experimentally measured changes in linoleic acid, lycopene, and hexanal with the concentration profiles predicted by the kinetic model, thereby evaluating the accuracy and applicability of the simulation. All analyses were carried out in triplicate for each experimental condition, and results are expressed as mean ± standard deviation (SD).

#### 2.2.2. Reagents and Standards

All solvents used for extraction and chromatographic analysis were of analytical or HPLC grade. Hexane, acetone, ethanol, methanol, acetonitrile, and ammonium acetate were purchased from Sigma-Aldrich (St. Louis, MO, USA). Sodium chloride (NaCl) used for headspace extraction was obtained from Merck (Darmstadt, Germany). A certified fatty acid methyl ester (FAME) standard mixture used for the identification of fatty acids, including linoleic acid, was obtained from Supelco (Bellefonte, PA, USA). Lycopene analytical standard (≥95% purity) used for calibration was purchased from Sigma-Aldrich (St. Louis, MO, USA). Ultrapure water used in chromatographic analyses was produced using a Milli-Q purification system (Millipore, Bedford, MA, USA). All solutions were prepared fresh when required and stored under appropriate conditions to minimize degradation of light-sensitive compounds such as carotenoids.

#### 2.2.3. Linoleic Acid Determination

Fatty acid composition was determined using a modified version of Solaberrieta et al. method, after lipid extraction and transesterification to fatty acid methyl esters (FAMEs) [31]. The analysis was performed according to AOCS Official Method Ce 1h-05 [32]. FAMEs were analyzed using an Agilent 6890N GC-FID (Agilent, Santa Clara, CA, USA) system equipped with a ZB-WAX capillary column (30 m × 0.25 mm × 0.25 μm). Helium was used as carrier gas at 1 mL min^−1^. The split ratio was 1:20 and the injection volume was 1 μL. The oven temperature program was 60 °C for 1 min, increased to 200 °C at 10 °C min^−1^ and held for 2 min, then increased to 220 °C at 5 °C min^−1^ and held for 20 min. Injector and detector temperatures were set at 250 °C. Linoleic acid was identified by comparison of retention times with those of a certified FAME standard mixture and expressed as percentage of total fatty acids.

#### 2.2.4. Lycopene Determination

A modified version of Montesano et al. was applied, briefly lycopene was extracted from 0.5 g of homogenized tomato pomace using 10 mL hexane:acetone:ethanol (2:1:1, *v*/*v*/*v*) [33]. After vortex mixing and standing for 10 min at room temperature, the extract was centrifuged at 11,000 rpm for 2 min and filtered through a 0.45 μm cellulose membrane filter. UHPLC-DAD (Thermo Fisher Scientific, Waltham, MA, USA) analysis was performed on a Vanquish system equipped with an Acclaim C30 column (3 μm, 3.0 × 150 mm) maintained at 40 °C. The mobile phase consisted of methanol containing 3.2 g L^−1^ ammonium acetate (70%) and acetonitrile (30%), delivered at 1.7 mL min^−1^. Detection was carried out at 460 nm and quantification was based on an external calibration curve prepared from certified lycopene standards.

#### 2.2.5. Hexanal Analysis

Hexanal, used as a marker of lipid oxidation, was determined by a modified version of Azarbad and Jeleri method that uses headspace solid-phase microextraction coupled with gas chromatography–mass spectrometry (HS-SPME-GC-MS) [34]. A 3 g portion of sample was placed in a headspace vial together with 3 g NaCl to enhance the release of volatile compounds into the headspace. The vial was sealed with a TFE–silicone septum and subjected to headspace extraction. Chromatographic analysis was performed using an Agilent 6890N (Santa Clara, CA, USA) gas chromatograph coupled to a mass spectrometer, equipped with an HP-5MS capillary column (60 m × 0.20 mm × 0.25 μm). Hexanal was identified by comparison of mass spectra with library databases and retention characteristics. Results were expressed as relative peak area.

### 2.3. Statistical Analysis

Statistical analysis was performed using Python (version 3.12) with the NumPy (version 1.24.4), Pandas (version 2.2.1), SciPy (version 1.13.0), and Statsmodels libraries (version 1.14.2). All measurements were carried out in triplicate, and results are reported as mean ± standard deviation (SD). Differences between treatments (fresh samples and samples heated at 50 °C, 70 °C, and 90 °C for 4 h and 12 h) were evaluated using two-way analysis of variance (ANOVA), considering temperature and time as independent factors. When statistically significant differences were observed, Tukey’s post hoc test was applied to identify differences between individual temperature treatments. Statistical significance was considered at *p* < 0.05. Data processing and graphical representation were performed using the Matplotlib (version 3.8.4) and Seaborn libraries (version 0.13.2). Detailed simulation outputs are provided in the Supplementary Materials (Table S1 and Text S1).

## 3. Results

### 3.1. Simulation Results

#### 3.1.1. Temperature Effect on Lipid Oxidation Dynamics

**Figure 1:**
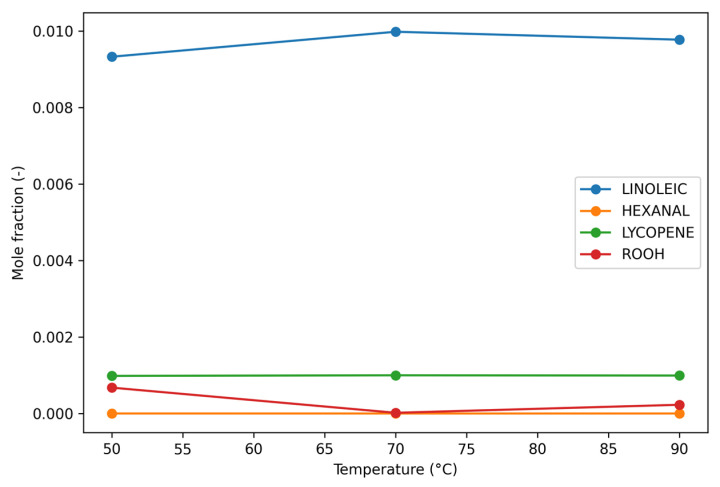
Temperature-dependent variation of simulated mole fractions of linoleic acid, hexanal, lycopene, and hydroperoxides (ROOH) after 12 h of reaction.

The simulated evolution of key species at different temperatures is presented in Table 3 and illustrated in Figure 1. The results indicate a clear temperature-dependent acceleration of lipid oxidation processes in tomato pomace.

At 50 °C, the system remained relatively stable, with minimal changes in the mole fractions of linoleic acid and lycopene after 12 h. Linoleic acid decreased only slightly from 0.010000 to 0.009982, corresponding to negligible oxidation, while lycopene exhibited a loss below 0.1%, indicating effective antioxidant protection under mild thermal conditions. Hexanal formation remained extremely low (8.21 × 10^−11^), confirming limited secondary oxidation. In contrast, increasing the temperature to 70 °C led to a noticeable intensification of oxidative processes. After 12 h, linoleic acid decreased to 0.009774, accompanied by a significant increase in hexanal formation (2.50 × 10^−8^). Lycopene degradation also became more pronounced, suggesting a partial reduction in antioxidant capacity. The results demonstrate a non-linear temperature dependence, with a transition from a kinetically limited regime at 50 °C to a strongly propagation-driven oxidation process at 90 °C. Two-way ANOVA showed that temperature had a statistically significant effect on linoleic acid degradation at both 4 h and 12 h (*p* < 0.05). Tukey’s post hoc analysis confirmed that linoleic acid loss at 90 °C was significantly higher than at 50 °C and 70 °C (*p* < 0.05), while differences between 50 °C and 70 °C were also statistically significant, although less pronounced.

#### 3.1.2. Reaction Pathway and Radical Dynamics

**Figure 2:**
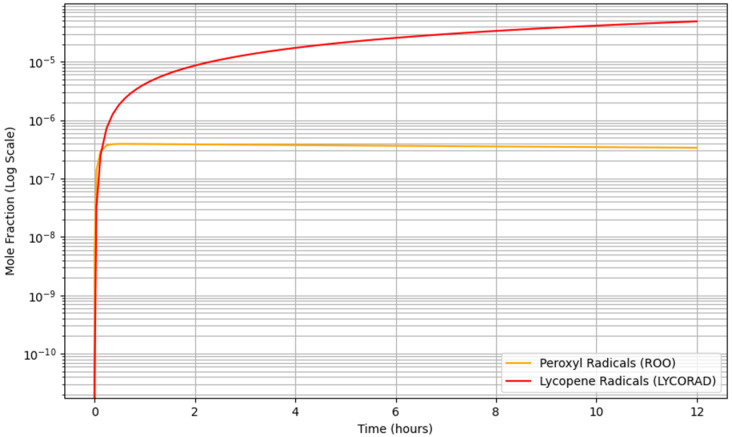
Time evolution of peroxyl radicals (ROO) and lycopene-derived radicals (LYCORAD) at 90 °C over 12 h, illustrating the rapid establishment of a quasi-steady-state for ROO and the continuous accumulation of antioxidant-derived radicals.

The simulation enabled the tracking of intermediate radical species, providing mechanistic insight into the oxidation process. The fastest elementary step across all temperatures was the oxygen addition reaction (LINRAD + O_2_ → ROO), indicating that oxygen incorporation represents the primary driver of the oxidation chain. The apparent rate of this reaction increased by approximately two orders of magnitude between 50 °C and 90 °C, confirming its strong temperature sensitivity. Hydroperoxides (ROOH) were identified as the dominant intermediate species. Their concentration increased from 1.84 × 10^−5^ at 50 °C to 1.83 × 10^−3^ at 90 °C after 12 h, representing an increase of approximately two orders of magnitude. This behavior indicates that hydroperoxides act as relatively stable reservoirs of oxidation, accumulating progressively during the propagation phase before undergoing thermal decomposition. The temporal evolution of radical species at 90 °C is presented in Figure 2. Peroxyl radicals (ROO) were rapidly formed during the initial stage of the reaction, reaching a quasi-steady-state concentration within the first hour of simulation. This behavior indicates that ROO radicals are highly reactive intermediates, continuously generated and consumed during the propagation phase of lipid oxidation. lycopene-derived radicals (LYCORAD) exhibited a continuous increase over the entire 12 h period, without reaching a plateau. This progressive accumulation reflects the ongoing participation of lycopene in radical scavenging reactions, leading to the formation of relatively more stable radical species. The divergence between the rapid stabilization of ROO and the sustained growth of LYCORAD suggests a dynamic balance between radical generation and antioxidant-mediated quenching. Hydroperoxides (ROOH) were identified as the dominant intermediate species in the system, with concentrations increasing by approximately two orders of magnitude between 50 °C and 90 °C after 12 h. This behavior indicates that hydroperoxides act as relatively stable reservoirs of oxidation, accumulating during the propagation phase before undergoing thermal decomposition into volatile secondary oxidation products such as hexanal.

#### 3.1.3. Oxygen Consumption and Oxidative Intensity

**Figure 3:**
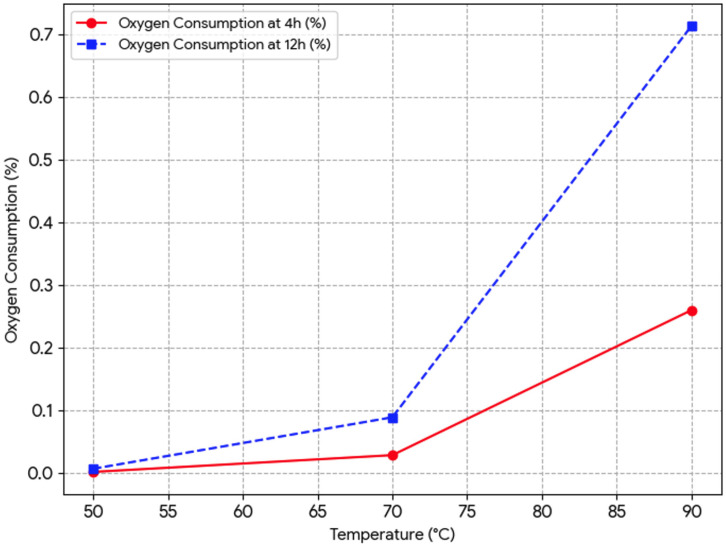
Simulated oxygen uptake in tomato pomace.

Oxygen consumption trends further confirm the temperature-dependent intensification of the oxidation process (Figure 3). At 50 °C, oxygen uptake remained minimal (0.007% after 12 h), indicating that the system is largely controlled by antioxidant activity and limited radical propagation. In contrast, at 90 °C, oxygen consumption increased to 0.714%, representing an approximately 100-fold increase compared to 50 °C. This substantial increase indicates a transition from a kinetically limited system to a propagation-dominated oxidation regime, where oxygen is rapidly consumed in radical reactions. These results demonstrate that thermal stress significantly enhances the catalytic cycle of lipid oxidation, leading to sustained radical generation and accelerated formation of secondary oxidation products.

#### 3.1.4. Selectivity of Oxidation

The relative degradation of linoleic acid and lycopene provides additional insight into the selectivity of the oxidation process. At 90 °C after 12 h, the ratio between linoleic acid loss and lycopene degradation was approximately 3.8:1, indicating that polyunsaturated fatty acids are significantly more susceptible to thermal oxidation than carotenoid compounds within the modeled system. This observation suggests that, although lycopene contributes to radical scavenging, its protective effect is insufficient to prevent extensive lipid oxidation under high-temperature conditions.

### 3.2. Model Validation

#### 3.2.1. Linoleic Acid Validation

The predictive capability of the kinetic model was evaluated by comparing the simulated linoleic acid loss with experimental measurements under identical thermal conditions (Table 4). Linoleic acid was selected as the primary validation marker due to its central role as a substrate in lipid oxidation and its direct representation within the reaction mechanism.

**Figure 4:**
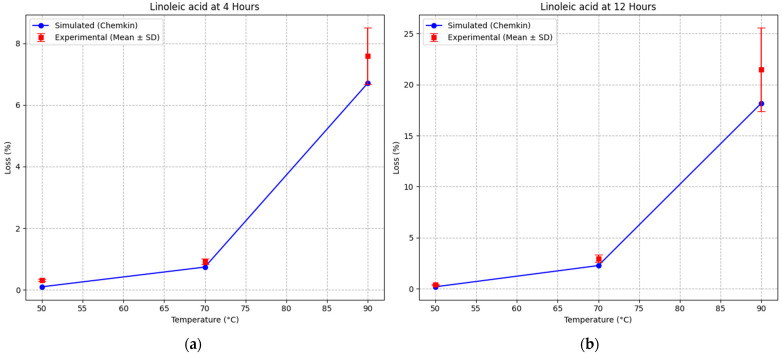
Comparison between simulated and experimental linoleic acid loss at (**a**) 4 h and (**b**) 12 h as a function of temperature. Experimental data are presented as mean ± standard deviation (*n* = 3).

At 4 h, the model reproduced the experimental trend with good accuracy across all temperatures. At 50 °C, the simulated linoleic acid loss (0.10%) was slightly lower than the experimental value (0.32 ± 0.04%), indicating minimal oxidation under mild thermal conditions. At 70 °C, the model predicted a loss of 0.74%, compared to 0.92 ± 0.10% experimentally, showing close agreement. At 90 °C, the simulated loss (6.71%) was in good accordance with the experimental value (7.59 ± 0.91%), confirming the model’s ability to capture the onset of accelerated oxidation. At 12 h, a similar level of agreement was observed. The model slightly underestimated oxidation at 50 °C (0.18% vs. 0.28 ± 0.07%), while at 70 °C it predicted a linoleic acid loss of 2.26%, compared to 2.96 ± 0.37% experimentally. At 90 °C, the simulated value (18.17%) closely matched the experimental result (20.46 ± 4.15%), remaining within the experimental variability range (Figure 4).

No statistically significant differences were observed between simulated and experimental values within the same temperature conditions (*p* > 0.05), indicating good agreement between model predictions and experimental data.

#### 3.2.2. Lycopene Validation

**Figure 5:**
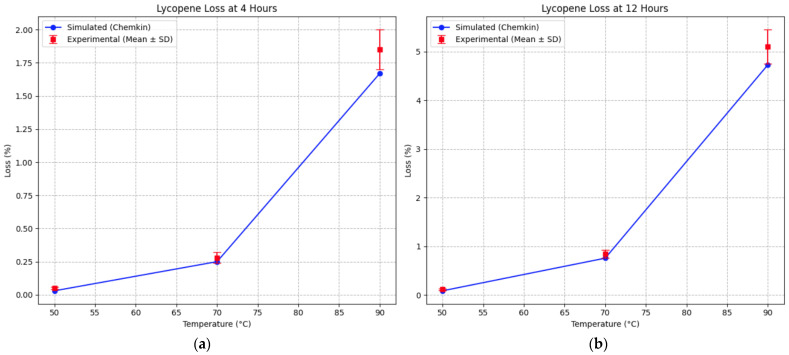
Comparison between simulated and experimental lycopene loss at (**a**) 4 h and (**b**) 12 h as a function of temperature. Experimental data are presented as mean ± standard deviation (*n* = 3).

The predictive performance of the model was further evaluated by comparing simulated and experimental lycopene loss under the same thermal conditions (Table 5, Figure 5). Lycopene was considered as a secondary validation marker due to its role as a natural antioxidant and its involvement in radical scavenging reactions within the proposed mechanism. At 4 h, the model reproduced the experimental trend across all temperatures with good accuracy. At 50 °C, the simulated lycopene loss (0.03%) was close to the experimental value (0.05 ± 0.01%), indicating minimal degradation under mild thermal conditions. At 70 °C, the predicted loss (0.25%) was in good agreement with the experimental value (0.28 ± 0.04%). At 90 °C, the model slightly underestimated degradation, predicting a loss of 1.67% compared to the experimental value of 1.85 ± 0.15%.

At 12 h, the model reproduced the experimental trend across all temperatures with good accuracy. At 50 °C, the simulated lycopene loss (0.09%) was close to the experimental value (0.12 ± 0.02%), indicating limited degradation under mild thermal conditions. At 70 °C, the predicted loss (0.76%) showed good agreement with the experimental value (0.85 ± 0.08%). At 90 °C, the model slightly underestimated degradation, predicting a loss of 4.73% compared to the experimental value of 5.10 ± 0.35%.

Statistically significant differences in lycopene degradation were observed between all temperature levels at both 4 h and 12 h (*p* < 0.05), confirming the strong temperature dependence of antioxidant depletion.

#### 3.2.3. Hexanal Validation

**Figure 6:**
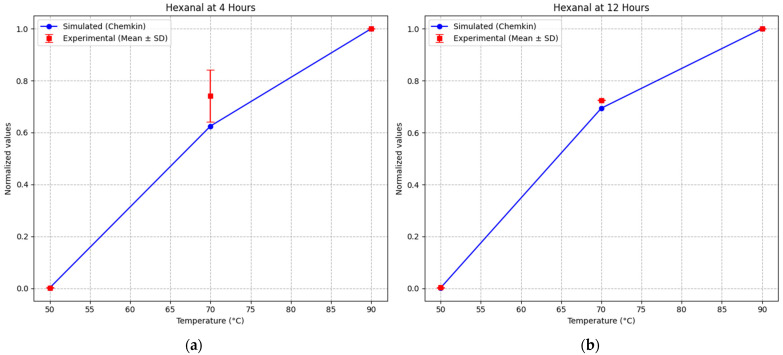
Comparison between simulated and experimental normalized hexanal formation at (**a**) 4 h and (**b**) 12 h as a function of temperature. Experimental data are presented as mean ± standard deviation (*n* = 3).

Hexanal formation was used as a secondary validation parameter to assess the model’s ability to predict the formation of volatile oxidation products. Due to differences in the nature of the data, simulated values (mole fraction) and experimental measurements (peak area) were converted to dimensionless normalized values. Normalization was performed by dividing each value by the maximum value obtained at 90 °C for the corresponding time point (4 h or 12 h), according to the equation:

(1) $$X_{norm}=\frac{X_{i}}{X_{max}}$$

where *X_i_* represents the measured or simulated value and *X_max_* is the maximum value within each dataset. This approach enables direct comparison of relative trends between simulated and experimental data independently of absolute units. (Table 6, Figure 6).

At 4 h, the model reproduced the temperature-dependent increase in hexanal formation with high accuracy. At 50 °C, both simulated and experimental normalized values were close to zero (0.0019 and 0.0021, respectively), indicating negligible oxidation. At 70 °C, the simulated value (0.625) showed good agreement with the experimental value (0.741), capturing the transition toward more active oxidation. At 90 °C, both simulated and experimental values reached the normalized maximum (1.0), confirming the strong oxidation regime at elevated temperature.

At 12 h, a similar trend was observed. At 50 °C, normalized hexanal values remained very low (0.0023 simulated and 0.0031 experimental), confirming limited oxidation. At 70 °C, the simulated value (0.694) closely matched the experimental value (0.725), demonstrating good predictive performance. At 90 °C, both datasets again reached the maximum normalized value (1.0), indicating that the model accurately captures the high-temperature oxidation regime.

#### 3.2.4. Statistical Validation

The agreement between simulated and experimental data was further quantified using statistical indicators, including the coefficient of determination (R^2^), root mean square error (RMSE), and mean percentage error (MPE) (Table 7).

The model exhibited a high level of accuracy for all evaluated parameters. For linoleic acid, the R^2^ value of 0.9761 indicates a strong correlation between simulated and experimental results, confirming that the model effectively captures the primary oxidation dynamics. The corresponding RMSE (0.5784) and MPE (23.52%) suggest moderate deviations, which are consistent with the slightly underestimated values observed at intermediate temperatures. For lycopene, the model showed improved predictive performance, with an R^2^ of 0.9899 and lower error values (RMSE = 0.2205; MPE = 14.28%). These results indicate that the model accurately describes the degradation of antioxidant compounds, although minor deviations remain at higher temperatures, likely due to additional thermal degradation pathways not included in the mechanism. The best agreement was obtained for hexanal, with an R^2^ value of 0.9982, indicating excellent correlation between simulated and experimental data. The low RMSE (0.0179) and MPE (10.03%) confirm that the model accurately captures the formation of secondary oxidation products and their strong dependence on temperature.

## 4. Discussion

The high correlation coefficients obtained for lycopene (R^2^ = 0.9899), hexanal (R^2^ = 0.9982), and linoleic acid (R^2^ = 0.9761) demonstrate that the Arrhenius-based multi-step mechanism effectively captures the thermal sensitivity of tomato pomace oxidation. However, the Mean Percentage Error (MPE) for linoleic acid (23.52%) was significantly higher than that of lycopene (14.28%). This discrepancy reflects the physical state of lipids within the pomace and highlights a structural limitation of the homogeneous reactor assumption. In the Chemkin model, linoleic acid is treated as a freely available reactant, whereas in real tomato pomace a substantial fraction is sequestered within the triacylglycerols of the seeds [35]. This matrix protection effect limits oxygen accessibility and reduces effective radical propagation rates compared to the idealized simulation environment. Consequently, oxidation in real systems operates under partially diffusion-controlled conditions, while the model assumes purely reaction-controlled kinetics. Similar observations were reported by Briones-Labarca et al., who demonstrated that the structural integrity of tomato by-products acts as a barrier to lipid oxidation during thermal processing [36].

The results show that lycopene degradation remains below 5% even after 12 h at 90 °C, confirming its effectiveness as a radical scavenger under the tested conditions. This behavior can be mechanistically explained by the preferential reaction of lycopene with peroxyl radicals, which reduces the effective propagation rate of lipid oxidation and delays the formation of secondary products. The observed stability is superior to results reported for tomato purée or juice, where lycopene loss can exceed 20% under similar temperatures [37]. This difference highlights the critical role of matrix structure, suggesting that intact pomace provides a diffusion barrier and a protective microenvironment not present in homogenized systems. In addition, the presence of co-antioxidants such as α-tocopherol in tomato seeds may further contribute to synergistic stabilization effects. Hexanal formation reached 11.29 µg/g at 90 °C after 12 h. According to Baenas et al., hexanal is a reliable marker for the secondary stage of lipid oxidation in omega-6 rich food matrices [38]. The excellent agreement between simulated and experimental data (R^2^ = 0.9982) confirms that Reaction 4 (ROOH → HEXANAL + FRAG12) accurately represents the dominant pathway of oxidative degradation in tomato lipids [39]. This consistency with literature-reported behavior supports the validity of the mechanistic framework used in the model.

The kinetic behavior of hydroperoxides warrants particular attention in the context of food quality prediction. In the present model, ROOH concentrations increased by approximately two orders of magnitude between 50 °C and 90 °C after 12 h, confirming their role as key transient intermediates in the autoxidation cascade. This accumulation–decomposition pattern is consistent with classical Bolland and Gee kinetics and with observations in bulk linoleic acid systems. Mechanistically, this behavior reflects the balance between formation through propagation reactions and depletion via thermal decomposition. At 50 °C, the low rate of initiation limits ROOH formation, while at 90 °C the rapid increase in radical flux leads to accelerated ROOH accumulation followed by decomposition into secondary volatiles. This transition has direct implications for food quality, indicating that oxidation progresses non-linearly and cannot be adequately described by simple first-order models at elevated temperatures [40]. A notable mechanistic feature of the proposed model is the explicit representation of two distinct lycopene scavenging pathways: direct quenching of peroxyl radicals (ROO + LYCOPENE → ROOH + LYCORAD) and chain-breaking regeneration of linoleic acid via lipid radical scavenging (LINRAD + LYCOPENE → LINOLEIC + LYCORAD). The latter interrupts radical propagation prior to oxygen addition, representing a kinetically significant protective mechanism consistent with the established chain-breaking activity of carotenoids. This dual scavenging mechanism explains the preferential protection of linoleic acid at 50 °C and 70 °C, where antioxidant capacity remains sufficient to suppress propagation. At 90 °C, however, the exponential increase in rate constants leads to a radical generation rate that exceeds the scavenging capacity of lycopene, resulting in a propagation-dominated regime. This kinetic saturation of antioxidant capacity represents a critical threshold for process optimization. Compared to alternative modeling approaches such as MATLAB-based ODE solvers [41] or empirical Arrhenius models [42], the Chemkin framework provides enhanced capability for resolving intermediate species and handling stiff reaction systems, offering a more robust platform for mechanistic modeling in food systems.

While the model demonstrates strong predictive performance, its interpretation must be contextualized within the structural complexity of real food systems. The Closed Homogeneous Reactor approach assumes perfect mixing and uniform phase distribution, whereas in tomato pomace lipids and antioxidants are compartmentalized within heterogeneous structures. These structural and diffusional constraints significantly influence oxidation kinetics and explain the systematic underestimation of linoleic acid degradation at intermediate temperatures. Incorporation of diffusion-reaction coupling and matrix accessibility parameters would likely improve predictive accuracy. Furthermore, the current mechanism does not account for additional antioxidant compounds such as phenolics and tocopherols, which may contribute synergistically to oxidative stability [24]. The thermodynamic properties of macromolecular species were estimated based on structural analogies, and further refinement through sensitivity analysis would improve model robustness. In addition, the use of hexanal as a single marker does not capture the full spectrum of secondary oxidation products, and future models should include a broader range of aldehydes to better represent sensory quality. This agreement indicates that, despite matrix complexity, the fundamental reaction pathway governing secondary oxidation remains consistent with previously reported omega-6 lipid systems [38,39].

The validation dataset, although showing strong agreement with the model, is based on a limited number of experimental conditions and selected markers. Therefore, the model should be interpreted as a mechanistic representation of intrinsic reaction kinetics rather than a complete description of real food systems. The linoleic acid degradation rate at 90 °C (18.17% over 12 h) falls at the lower end of values reported for thermally treated tomato systems (20–35%), consistent with the matrix protection effect discussed above. This contrasts with homogenized systems reported in the literature [37], where the absence of structural barriers increases oxygen accessibility and accelerates carotenoid degradation. The higher stability of lycopene compared to tomato purée or juice [37,39,40] further supports the role of structural organization in modulating oxidation behavior. Compared to emulsified or bulk systems, oxidation in tomato pomace is partially diffusion-limited, resulting in slower effective degradation rates despite similar thermal conditions.

From an applied perspective, the identification of a kinetic threshold between 70 °C and 90 °C provides a mechanistically grounded basis for process optimization. Above this threshold, rapid hydroperoxide decomposition and secondary oxidation lead to accelerated quality deterioration. This suggests that milder thermal treatments or short-time high-temperature processes are preferable to prolonged exposure at intermediate temperatures. In industrial applications such as drying or pasteurization, the model can be used to estimate cumulative oxidative damage based on time–temperature profiles, supporting optimization of residence time and oxygen exposure. The applicability of the model is primarily limited to conditions where thermal effects dominate over structural transformations, such as drying, pasteurization, or intermediate moisture systems. In more complex matrices, additional factors including phase partitioning and diffusion gradients must be considered. Therefore, the model should be regarded as a baseline representation of intrinsic chemical reactivity, which can be extended through incorporation of matrix-specific parameters. In practical terms, the model can be used as a predictive tool to support process optimization by identifying safe operating temperature ranges, estimating oxidation risk under different processing scenarios, and guiding the design of thermal treatments prior to experimental validation.

Beyond its application to tomato pomace, this study demonstrates the feasibility of applying reaction engineering tools traditionally used in combustion chemistry to food systems governed by radical-mediated processes. The integration of mechanistic modeling with experimental validation represents a step toward predictive food process engineering, enabling a transition from empirical approaches to model-driven optimization. This approach is particularly relevant for the valorization of agro-industrial by-products, where variability in composition and processing conditions requires robust predictive tools.

## 5. Conclusions

This study demonstrates that lipid oxidation in tomato pomace can be effectively described using a simplified kinetic model implemented in Chemkin. The results confirm a strong temperature dependence of oxidation processes, with a transition from a kinetically limited regime at 50 °C to a propagation-dominated system at 90 °C. Linoleic acid was identified as the most sensitive component to thermal oxidation, while lycopene exhibited higher stability due to its radical scavenging activity. The model successfully reproduced experimental trends for all validation parameters, showing strong agreement for linoleic acid and lycopene and excellent predictive accuracy for hexanal formation. The accumulation of hydroperoxides and their subsequent decomposition into volatile compounds were identified as key steps governing oxidation intensity. From an applied perspective, the results indicate that under the controlled conditions investigated in this study, temperatures above 70 °C were associated with accelerated oxidation, suggesting that lower processing temperatures may be beneficial for preserving bioactive compounds in tomato pomace. Therefore, controlling thermal conditions is essential to preserve the nutritional and sensory properties of tomato pomace-derived products. In this context, the proposed model provides a useful framework for supporting decision-making in the design and optimization of food processing operations involving lipid-rich by-products. Future studies should focus on a narrower temperature range (70–80 °C) to better identify the onset of lycopene degradation and support optimization of processing conditions for improved retention of bioactive compounds.

## Figures and Tables

**Table 1:** Initial species composition used in the kinetic simulations.

Species	Mole Fraction
N_2_	0.779
O_2_	0.210
Linoleic acid	0.010
Lycopene	0.001

**Table 2:** Reaction mechanism for lipid oxidation and lycopene antioxidant activity used in the kinetic model.

Step Type	Reaction Equation	Pre-Exponential Factor (cm^3^·mol^−1^·s^−1^)	Activation Energy (cal·mol^−1^)	Description
Initiation	LINOLEIC + O_2_ → LINRAD + HO_2_	1.0 × 10^13^	24,500	Formation of lipid radicals initiating the oxidation chain.
Oxygen addition	LINRAD + O_2_ → ROO	1.0 × 10^12^	0	Rapid formation of peroxyl radicals (near collision limit).
Propagation	ROO + LINOLEIC → ROOH + LINRAD	1.0 × 10^14^	14,000	Hydrogen abstraction leading to hydroperoxide formation and chain propagation.
Hydroperoxide decomposition	ROOH → HEXANAL + FRAG12	1.0 × 10^14^	35,000	Thermal breakdown of hydroperoxides into secondary oxidation products.
Antioxidant (ROO scavenging)	ROO + LYCOPENE → ROOH + LYCORAD	1.0 × 10^11^	10,000	Neutralization of peroxyl radicals by lycopene.
Antioxidant (LINRAD scavenging)	LINRAD + LYCOPENE → LINOLEIC + LYCORAD	1.0 × 10^10^	10,000	Regeneration of lipid and inhibition of radical propagation.

**Table 3:** Simulated mole fractions of linoleic acid, hexanal, and lycopene at different temperatures and reaction times.

Timepoint	Temperature °C	Linoleic Acid Mole Fraction (-)	Hexanal Mole Fraction (-)	Lycopene Mole Fraction (-)	ROOH Mole Fraction (-)
0 h	-	0.010000	0	0.0010000	0
4 h	50	0.009990	7.93 × 10^−12^	0.0009997	5.69 × 10^−6^
	70	0.009926	2.63 × 10^−9^	0.0009975	7.45 × 10^−5^
	90	0.009329	4.21 × 10^−7^	0.0009833	6.76 × 10^−4^
12 h	50	0.009982	8.21 × 10^−11^	0.0009991	1.84 × 10^−5^
	70	0.009774	2.50 × 10^−8^	0.0009924	2.28 × 10^−4^
	90	0.008183	3.60 × 10^−6^	0.0009527	1.83 × 10^−3^

**Table 4:** Simulated and experimental linoleic acid loss percentage.

Timepoint	Temperature °C	Simulated Linoleic Acid Loss (%)	Experimental Linoleic Acid Loss (%)
4 h	50	0.10	0.32 ± 0.04 ^a^*
	70	0.74	0.92 ± 0.10 ^b^
	90	6.71	7.59 ± 0.91 ^c^
12 h	50	0.18	0.28 ± 0.07 ^a^
	70	2.26	2.96 ± 0.37 ^b^
	90	18.17	20.46 ± 4.15 ^c^

* Different superscript letters (a–c) within the same timepoint indicate statistically significant differences between temperature treatments (*p* < 0.05) according to Tukey’s post hoc test.

**Table 5:** Simulated and experimental lycopene loss percentage.

Timepoint	Temperature °C	Simulated Lycopene Loss (%)	Experimental Lycopene Loss (%)
4 h	50	0.03	0.05 ± 0.01 ^a^*
	70	0.25	0.28 ± 0.04 ^b^
	90	1.67	1.85 ± 0.15 ^c^
12 h	50	0.09	0.12 ± 0.02 ^a^
	70	0.76	0.85 ± 0.08 ^b^
	90	4.73	5.10 ± 0.35 ^c^

* Different superscript letters (a–c) within the same timepoint indicate statistically significant differences between temperature treatments (*p* < 0.05) according to Tukey’s post hoc test.

**Table 6:** Simulated and experimental normalized hexanal values at different temperatures and reaction times.

Timepoint	Temperature °C	Simulated Normalized Values Hexanal	Experimental Normalized Values Hexanal
4 h	50	0.0019	0.0021 ^a^*
	70	0.625	0.741 ^b^
	90	1	1 ^c^
12 h	50	0.0023	0.0031 ^a^
	70	0.694	0.725 ^b^
	90	1	1 ^c^

* Different superscript letters (a–c) within the same timepoint indicate statistically significant differences between temperature treatments (*p* < 0.05) according to Tukey’s post hoc test. Values were normalized relative to the maximum value at 90 °C for each timepoint.

**Table 7:** Statistical validation of Chemkin simulation vs. Experimental data.

Parameter	Linoleic Acid	Lycopene	Hexanal
R-squared (R^2^)	0.9761	0.9899	0.9982
Root Mean Square Error (RMSE)	0.5784	0.2205	0.0179
Mean Percentage Error (MPE)	23.52%	14.28%	10.03%
Fit Quality	Strong	Good	Excellent

## Data Availability

The original contributions presented in this study are included in the article/Supplementary Materials. Further inquiries can be directed to the corresponding authors.

## Supplementary Materials

The following supporting information can be downloaded at: https://www.mdpi.com/article/10.3390/foods15091522/s1, Table S1: Simulation results at 4 h and 12 h. Text S1: Thermal data of the reactions.
